# “All Hands on Deck”: A Systematic Review of Concussion Guidelines Across All Sailing Sports and a Call to Action

**DOI:** 10.3390/sports13120455

**Published:** 2025-12-18

**Authors:** Isabelle Graham, Ffion Taylor, Neil Heron

**Affiliations:** 1 Centre for Public Health, Queen’s University Belfast, Belfast BT7 1NN, Northern Ireland, UK; 2 Medical Department, SailGP, London SW7 4ES, UK

**Keywords:** sailing, concussion, head injuries, protocols

## Abstract

**Background:** Sports-related concussions are complex, traumatic brain injuries as a result of a sporting accident. Prompt diagnosis and assessment with the use of diagnostic protocols help provide athletes with the appropriate management to minimise acute and chronic implications. **Objective:** The objective of this systematic review is to review the current sailing concussion assessment and diagnostic guidelines and consequently propose a sailing concussion assessment and diagnostic protocol to use. **Methods:** Sailing organisations such as “World Sailing” were used to find current guidelines used in the sailing community. Electronic databases such as Google Scholar, Springer Link, and PubMed were used to identify relevant scientific papers. Keywords included “sailing”, “concussion”, “guidelines”, “sailing legislation”, and “sports-related concussion”. This systematic review is not limited to a specific sailing subtype. We included papers discussing concussion guidelines and excluded studies with no relevance to sports related concussions and without relevant guideline use. Findings were summarised in in text and tables. **Results:** While there is much research discussing head injuries in sailing, there is no literature specifically discussing protocols and guidance for concussion diagnosis and management in sailing-related concussions. Six concussion protocol papers are discussed. The World Sailing website advises individuals to use the CRT5 guidelines to assess a suspected concussion, which have been outdated by CRT6. While there are currently no standardised sailing-specific concussion assessment tools available, this review proposes a possible approach, introducing the concussion protocol used in the professional sailing league, SailGP. A sport-specific protocol is vital in addressing the specific risks associated with a sailing-related concussion. **Conclusions:** Concussions are a considerable risk in sailing due to the unique nature of the sport. Due to the lack of standardised concussion guidelines within the sailing community, a standardised, sport-specific concussion assessment tool, such as the one described for SailGP, should be developed through collaboration between medical professionals and sailing organisations.

## 1. Introduction

**Sports-Related Concussions**

Sports-related concussions (SRC) are complex, traumatic injuries to the head resulting in a functional injury of the brain [1]. These sports-induced head injuries present with a range of symptoms, such as headaches, nausea, and dizziness. Direct or indirect trauma to the body can transmit a force to the head, leading to a concussion, and therefore concussions can occur without an obvious head injury. The symptoms of a concussion can present within minutes to hours and typically resolve spontaneously over hours to days. Prompt identification of a concussion is essential to optimise treatment, as, although most concussions resolve spontaneously, there is a small risk of long-lasting complications which can impact daily life. Accurate identification of SRC is key to an athlete’s short- and long-term mental and physical health, as failure to recognise a concussion can result in an increased risk of SRC recurrence, and further physiological harm [2]. Most athletes experiencing SRC are not at risk of chronic effects. However, some studies highlight an association with cognitive deficits later in life following repeated concussions, illustrating the importance of correct management and care prior to return-to-play decisions [3].

As stated, although chronic effects following an SRC are not common, there are some short- and long-term risks of mismanaging concussions. The most common effect of the acute mismanagement of an SRC is the prolongation of the concussion symptoms from a short-term to long-term issue, which can even turn into post-concussion syndrome [4]. Studies indicate that most patients with post-concussion syndrome had a history of recurrent prior concussions or experienced a “double hit” [5]. Further long-term risks of poorly managed concussions include chronic traumatic encephalopathy, which is a neurodegenerative disease thought to be linked to repetitive head trauma [6].

Naturally, most sports involving a certain element of velocity are a risk factor for SRC. Sailing, in particular, poses a risk, with approximately 14% of injuries in sailing estimated to be concussions [7]. Commonly, sailing-related concussions occur as a result of impacts to the body or head from the boom or are caused by falls during manoeuvres [8]. However, due to the nature of the sport, there is often a delay in medical attention, as a result of the time it takes to reach land, and therefore prompt diagnosis and management of the concussion is often delayed. Furthermore, studies indicate that head injuries, including concussions, are more common in novice and intermediate sailors, who may have greater difficulty operating a boat whilst bringing the affected athlete to land [9]. Furthermore, there is an added significance to concussions in water sports, which can prove fatal due to the risk of drowning as a consequence of impaired consciousness in water [10]. Reports illustrate that drowning is the biggest cause of boating deaths, with 73.1% of mortalities being attributed to this [10].

**What is Sailing?**

Sailing is a water sport utilising wind and sails to propel a boat/vessel through the water. It can be viewed as a competitive sport, a means of transport, or a recreational activity. However, in this paper it will be considered as a competitive sport. There are different subtypes of competitive sailing, including fleet racing, match racing, team racing, offshore and oceanic sailing, and cruising [11].

Different sailing subtypes utilise specific sailing vessels. There are a number of different types of sailing vessels, the main ones being dinghy and keel boats. Each boat requires different skills, affecting different parts of the sailor’s body. The smallest sailing boat is the dinghy, with a length as small as 2.4 m, relying on one athlete to move the vessel without capsizing. In contrast, keel boats can reach up to 30 m in length, with a maximum of 22 sailors on board, each adopting different roles, dictating the position of the athlete on the vessel. Activities primarily include pulling lines and turning winches. Acknowledging the differences in these vessels, and the skills and roles required to sail them, is essential when discussing sailing injuries. For example, the most common injury regions in keel boat sailing are to the upper extremities, whilst in dinghy boats, the lower extremities, followed by the upper extremities and head/neck, are most likely to be injured [12]. Therefore, the difference in concussion occurrence across the different sailing subtypes emphasises the relevance of vessel type in the discussion of concussions in sailing [12].

**SRC Assessment**

To help assess SRC, the ‘Concussion In Sport Group’ (CISG) have published a number of Sport Concussion Assessment Tools (SCAT). This involves symptomatic evaluation, cognitive screenings, coordination and balance examinations, and delayed recall ability [13]. SCAT6 also incorporates Maddock’s Questions, which help assess an athlete’s memory and orientation. World Sailing uses the Concussion Recognition Tool (CRT5), published in 2017, to help identify concussions at sailing events. It is a four-step assessment tool which outlines red flags (when to contact an emergency service), signs and symptoms of a concussion, and a memory assessment [14]. However, while it is an established protocol, the tools used to assess concussions (SCAT and CRT) are not sport-specific and, therefore, need to be adapted to the specific demands of sailing.

The Concussion in Sport Group (CISG) continuously updates statements on the management of SRC, with the most recent update published in 2023, having been provisionally released and discussed in 2022. These guidelines are built on following the “11 R’s” of SRC to help map a logical management outline: Recognise, Reduce, Remove, Refer, Re-evaluate, Rest, Rehabilitate, Recover, Return-to-learn/Return-to-sport, Reconsider, and Residual Effects [15]. Adding a further two “R’s”, “Retire and Refine”, was proposed at the Amsterdam 2022 Statement to help address athletes’ career-changing decisions, which underlines the need to keep guidelines up to date [15]. It is crucial to analyse current concussion guidance in sailing to ensure that it is consistent with this “11R’s” approach.

## 2. Objective

This paper aims to review the current sailing concussion guidelines as well as to summarise the key findings in identification methods for concussion diagnosis. Furthermore, a sailing concussion policy (proposed by the professional sailing league, SailGP) will be described.

## 3. Materials and Methods

A systematic review was undertaken to synthesise and contextualise existing guidelines and protocols for concussions in sailing. Additionally, this review aims to aid in the identification of inconsistencies and limitations in current guidelines.

**Search Strategy**

Relevant articles and guidelines included in this systematic review were published before 12 May 2025. The articles were identified using the following electronic databases: Google Scholar, PubMed, and SpringerLink. Keywords searched in the electronic databases included: “sailing concussion”, “concussion”, “guidelines”, “sailing legislation”, and “sports-related concussion”.

The quality and relevance of the included articles were assessed by the credibility of the guideline applied and the relevance of it to sports-related concussions. Articles produced by official sailing and sporting organisations with transparent guideline use were prioritised. We extracted data on the following outcomes: concussion recognition criteria, recommended assessment tools and return-to-play protocols. A narrative synthesis was conducted. The possibility of reporting bias was assessed by cross-checking guideline across multiple organisations to identify missing or inconsistent aspects. Due to the qualitative nature, a statistical assessment was not possible. This systematic review followed the guidelines set by the Preferred Reporting Items for Systematic Reviews and Meta-Analyses (PRISMA) [16]. The completed PRISMA checklist is available in Supplementary File S1.

**Eligibility Criteria**

Inclusion criteria:

- Studies discussing concussion assessment guidelines in sailing sports or comparable sports;
- Sailing-specific articles, including guidelines, protocols, and policies;
- Articles published on reputable sites. Reputable sources include peer-reviewed articles from well-established scientific and medical journals, ensuring that the review article is based on credible medical literature.

Exclusion criteria:

- Opinion pieces, editorials, and magazines;
- Articles published in a non-English language;
- Lack of access to the full-text article;
- Articles discussing non sport related concussions.

## 4. Results

**Figure 1:**
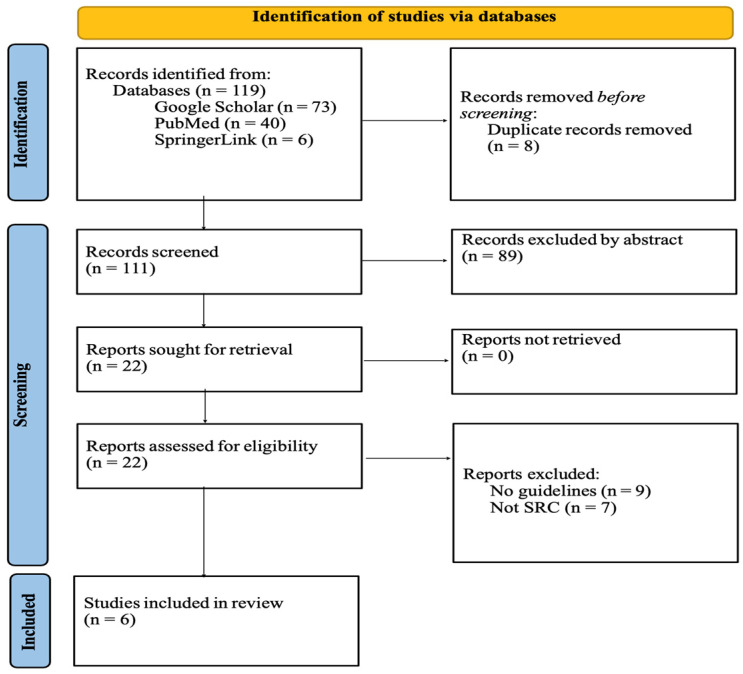
PRISMA 2020 flow diagram for new systematic review process.

22 papers were initially identified with the selected keywords as shown in Figure 1. I.G. and F.T. independently screened these for relevance and against the eligibility criteria. No automation tools were used. These papers then underwent full-text screening, with six papers included in this systematic review (Table 1). Extracted information was cross-checked by the authors. Most papers were removed from the systematic review due to their irrelevance to sports-related concussions, as well as a lack of discussion on the identification and management and use of protocol in an acute concussion.

**Concussion Guidelines in Sailing**

The “World Sailing” and “The Royal Yachting Association” guidance for concussion identification is to use the SRC recognition tool: CRT5 [14]. Additionally, clinical assessment is to be performed by a medical professional, as per SCAT5 guidelines [13].

**Return to Sail Protocol**

Although there are no sailing-specific, standardised guidelines for initial concussion assessment and management, there are guidelines in place for the return to sailing post-concussion practiced by international sailing organisations. The Royal Yachting Association (RYA) has published a six-stage “Pathway to Recovery” for the management of an athlete returning to sailing following a concussion [17].

## 5. Discussion

**Current Guidelines in Context of Relevant Literature and their Application**

The current guidelines adopted by World Sailing were determined by the “Consensus Statement on Concussion in Sport” in 2016, in the form of CRT5 [2]. CRT5 is a concussion identification tool designed for laypersons to quickly assess a possible concussion in four steps. SCAT6 is a more comprehensive concussion assessment tool, specifically used in sports by healthcare professionals to help assess suspected concussions. Both tools are intended for use in the acute phase of a suspected concussion. Additionally, by completing a baseline SCAT6 score, a post-injury SCAT6 score’s significance may be interpreted more accurately. They state that any athlete who has a suspected concussion should be removed from play, assessed, and monitored. Basic first aid principles should also be followed in an acute injury situation. Neither tool is sport-specific, and both are used in a wide variety of sports. Additionally, the only document found containing official guidance on concussion in sailing was the CRT5, published in 2017 [14].

The Concussion in Sport Group (CISG) has since updated their guidelines, in 2022 (CRT6). CRT6 is built on the same principles as CRT5; however, it includes further detail in areas such as athletes’ long-term effects and retirement. CRT5 and CRT6 are aimed for use by laypersons for a sideline evaluation of memory and cognitive function to determine the severity of a traumatic head injury. If red flags are present, management is escalated to emergency medical services. It also provides advice regarding athletes with a suspected or confirmed SRC and recommends that they be re-evaluated on a regular basis in the initial hours to days following the incident [15].

The guidelines include symptoms and signs of possible concussions, such as difficulty concentrating, drowsiness, and dizziness, as well as questions to aid in memory assessment, followed by advice for the affected athlete. Questions used in memory assessment include: “Where are we today?” and “What event were you doing?”.

However, even though these guidelines are not standardised within the sailing community, there is evidence of improved outcomes and reduced risk of further concussions if an athlete is promptly assessed, diagnosed, and managed [18]. For example, other sports with increased SRC risk, such as rugby, have demonstrated an improvement in concussion management since the implementation of concussion assessment protocols [19]. These guidelines have not been adapted for use in sailing. Furthermore, the positive results of a sport-specific concussion assessment tool are evident. For example, world rugby uses a three-stage rugby-specific Head Injury Assessment Protocol to assess a potential concussion, and since its implementation, there has been a significant decrease in rugby players returning to play with a concussion [20,21].

**Developing Sport-Specific Guidelines**

To develop a sport-specific concussion protocol, an evidence-based and clearly structured approach is necessary. Initially, a clear sporting population (in this instance: sailing) is identified, along with the risks associated with the sport. Following this, a thorough review of the epidemiology of concussions in the chosen sport, as well as the mechanism of these, should be undertaken to enable a suitable protocol design built on the gaps in prior implemented guidelines. The key components which the protocols need to consider are as follows: any concussion reporting procedures already used in international sporting bodies, sideline assessment tools (SCAT) with an additional sport-specific aspect, and clearly highlighted red flag features, as well as a stepwise return-to-play component. Furthermore, once a sport specific concussion protocol has been accepted by sporting organisations, concussion prevention strategies, such as education and rule enforcement, should be put in place alongside the protocol. To assess the effectiveness of the protocol, regular reviews should take place to ensure that any new findings can be integrated into the protocol and, as a result, improve athlete outcomes.

**Return to Play**

Return to Play programmes aim to prevent further injury for athletes returning to sport post-injury. These are multistep processes focusing on proper rehabilitation and fitness care prior to sport re-entry. Certain studies illustrate a reduction in injury risk following injury prevention programmes across multiple sports; however, there is a limited amount of available research on sailing-specific programmes [22].

Although there is no sailing-specific sailing concussion protocol, the RYA has published a sailing-specific “Pathway to Recovery” tool following a concussion [17].

The “Pathway to Recovery” tool describes a six-stage process, describing physical and cognitive progressions. These stages help guide athletes to a safe return to sailing following a concussion [17].

The first stage of the RYA pathway to recovery is on days 0–3 of a concussion, where total rest should be practiced. This includes abstaining from any forms of strenuous physical activity and taking time off work/school. Stage two occurs on days 2–6, where a slow reintroduction of day-to-day activities can begin. This includes activities such as reading and cooking, and light physical activity may take place. By days 7–9, stage three commences, and the individual can start to carry out work/school related tasks with regular breaks. Additionally, periods of physical activity can increase slightly, to approximately 15 min sessions. Stage four on days 10–14 includes a full return to work/school with either reduced working hours or increased break times. Stage five is approximately two weeks following the initial concussion, and the athlete can return to sailing, following a seven-phase sail protocol outlining different sailing sessions as well as a range of sailing intensities. Once the return to sail protocol has been completed, stage six is achieved, which considers the athlete to be back to their baseline health.

**Red Flags**

The concussion assessment tools (CRT and SCAT) also provide a list of red flags to assess a more severe form of spinal or traumatic brain injury:

- Neck pain/tenderness;
- Seizure/convulsion;
- Double vision;
- Loss of consciousness;
- Weakness/tingling/burning in 1+ arm/leg;
- Deteriorating conscious state;
- Vomiting;
- Severe/increasing headache;
- Increasingly restless/agitated/combative;
- GCS < 15;
- Visible deformity of skull.

In cases of a red flag symptom presentation, urgent medical attention should be prioritised, with the athlete immediately removed from the field of play. Red flag conditions associated with concussions include the following: cervical spine injury, intracranial injuries, subdural hematomas, epidural hematomas, cerebral contusions, intracerebral hematomas, or haemorrhages [23]. Subdural hematomas are the leading cause of death in sports related head injuries and therefore should be considered in any potentially affected individual [24].

**Unique Concussion Risks in Sailing**

Despite the lack of literature available for concussion guidelines in sailing, there are a number of papers discussing injuries in sailing and highlighting the severity of head injuries and concussions in sailing, even stating that head injuries are “the most life-threatening injury” due to “loss of consciousness” and “drowning” [25]. Additionally, “direct impact to the head” causing concussions was “the most common cause of medical evacuation from ship”, with some papers urging for the use of protective headgear when sailing [26]. However, due to the nature of concussions, helmets are thought to contribute little in the prevention of a concussion, as they do not lessen the rotational forces of head impacts, and only work to potentially reduce other head injuries such as lacerations [27]. Due to the significant risk of head injuries and concussions, sailing organisations have increasingly discussed which preventative measures should be in place to decrease the number of head injuries and concussions.

Although preventative measures may help to decrease the number of sailing-related concussions, due to the nature of the sport, concussions are still a risk, emphasising the need for a sailing-specific concussion protocol.

Sailing is a sport with a unique environment, and therefore certain risks. Environmental risk factors include unpredictable waves and wind conditions, leading to boat instability and, in turn, increasing the risk of falls, as well as the dangers of nighttime sailing [28]. Head trauma and concussions are some of the most severe injuries that occur in sailing due to their associated balance and coordination issues, risk of secondary injury, delayed reaction time, and environmental exposure [10]. Additionally, the boom of the boat is mobile and may unexpectedly swing as a result of wind or direction changes, posing a potential injury risk. Boom strikes are some of the most common causes of head injuries and concussions in sailing [10]. Furthermore, the crew can be offshore for a prolonged period, leading to delayed medical attention in case of an injury. Delayed removal from activity following concussion symptoms is linked with longer recoveries and further neuronal stress [29].

Aside from environmental risks, which pose the most significant risk factor for an acute injury, there is also a considerable physical factor involved with the sport. Sailing demands a high degree of balance, coordination, and concentration from athletes. A deficit in any of these can result in a range of injuries, including concussions. Therefore, despite the extrinsic injury risk factors, intrinsic risk factors such as poor crew communication, inadequate attentiveness, poor fitness, sleep deprivation, and lack of experience are linked to higher rates of injury incidences [7]. Additionally, the risk of drowning must also be considered in an unwell or injured athlete, especially if they are disorientated, dizzy, or have lost consciousness due to a concussion, and therefore all athletes are strongly encouraged to wear life jackets whilst sailing [25].

Differential diagnoses of concussions should also be excluded, as the unique aspects of sailing can predispose athletes to further medical conditions such as dehydration or seasickness. Differentiation is paramount, as symptoms overlap, and management can vary. An athlete struggling with seasickness, for example, may present with symptoms of dizziness, nausea, and fatigue, similar to symptoms of concussion. This further highlights the potential benefits of sailing-specific concussion guidelines.

**Limitations in Non-Standardised Sport-Specific Concussion Guidelines**

Although sports-specific concussion guidelines have the potential to address individual sport concussion risks and assessments, there may also be accompanying limitations, hence the importance of standardisation. Inconsistencies across sport guidelines may result in confusion and inconsistent application, posing a potential risk to athletes [30]. Moreover, some experts advocate for standardisation of concussion protocols. This would result in more evidence-based protocols, due to broader applicability and more frequent use. A specialised protocol carries the risk of inconsistent implementation due to limited training for staff. Therefore, a hybrid approach of sport-specification and standardisation should be implemented to ensure appropriate consideration of the niche requirements of specific sports and to ensure that standardised, evidence-based assessment guidelines are available. Therefore, guidelines should be based on, and need to reference, the key underlying sports concussion guidelines, making it clear which additional aspects should be focused on and considered when applying these to a specific sport.

**SailGP—A Proposed Concussion Management Tool (Appendix A)**

This article has highlighted the significant gap in sailing-specific concussion assessment tools. The SailGP on-water concussion assessment tool is a guideline specifically focusing on and outlining the acute assessment of sailing-related concussions within this professional sailing league and is based on the consensus statement on concussion in sport [15]. The protocol is aimed at medical, physiotherapy, paramedical, and coaching staff during competitions and training sessions, and should be applied to any athlete who may be affected. The Policy defines what concussions are, their significance, as well as the importance of recognising and taking action if a concussion is suspected. It aims to clearly discuss the prevention, identification, and management of a sailing-related concussion.

A list of symptoms and signs is provided for prompt identification of a concussion, such as loss of consciousness, impaired balance, and confusion. Furthermore, Sail-GP touches on the graduated return to full training (GRFT), which is a programme broken down into steps for the progression to returning to sailing following a concussion. The return to training should be guided by a medical team and should consider any necessary adjustments relevant to the athlete following their concussion. Additionally, the Sail-GP concussion policy touches on what an athlete should do if they experience persistent symptoms. It states that should an athlete experience symptoms beyond 10–14 days, they should seek a comprehensive medical assessment. This assessment typically identifies issues related to cervical dysfunction, vestibular–ocular–motor dysfunction, and mood and/or anxiety disorders. Depending on the extent of the issue, external specialist input and brain imaging, when necessary, may be required. Interventions for these issues include improving neck muscle strength, activating neck muscles prior to sailing, and vestibular–ocular–motor training to enhance balance in athletes.

The SailGP On-Water Concussion Assessment is broken down into nine steps. Step 1 is “Observable Signs” such as “facial injury after head trauma” or “lying motionless on the playing surface”, which are either present (yes) or absent (no). Step 2 is the “Glasgow Coma Scale” (GCS), which is a neurological assessment tool used to determine a patient’s consciousness level. It can total up to the highest score of 15, indicating full consciousness and responsiveness with no impairment, or the lowest possible score of 3, indicating a deep level of unconsciousness, which is associated with a high mortality rate and very poor prognosis [31]. Consciousness must be assessed with an added level of urgency in any water sports due to the risk of drowning with even a transient loss of consciousness [10]. This was exemplified in the 2023 fatality in the Lincoln Week Regatta, reported via an incident report published by “Australian Sailing”. Andrew McLeod, considered an “experienced and highly competent sailor”, died when he lost consciousness after a gybe, sustaining a large, closed head injury and falling under the lifeline, overboard [32].

Step 3 is “Cervical Spine Assessment”, which poses its own unique challenge on board a vessel, with a cervical spine injury requiring immobilisation and up to five people to transfer a patient from place to place. Thus, this must be prioritised and coordinated carefully while sailing. Step 4 is a “Coordination and Ocular/Motor Screen”, which are both dichotomous assessments essential in a safety assessment of an injured person on board a ship, with impaired balance and coordination potentially resulting in a ‘man overboard’ emergency.

Step 5 is “Memory Assessment Maddock’s Questions”. Maddock’s Questions are used to assess possible concussions in a nonclinical setting. They are a set of questions designed to evaluate the athlete’s orientation and recent memory. For example: “What race are we doing now?”. Following this, Step 6 is “Symptom Evaluation”, where common symptoms found in concussions are compared to the athlete’s presentation. Step 7, “Orientation”, is a further cognitive screen, which asks further detailed questions regarding the time and day, Step 8, “Concentration”, assesses the athlete’s cognitive function by asking them to recite the months of the year in reverse order. Finally, Step 9, “Delayed Recall”, is a list of random words, such as “jacket, arrow, pepper, cotton, movie”, mentioned earlier on in the assessment which the athlete is asked to recall at the end of the assessment. The cognitive effects of concussion can have a lasting impact on life at sea, especially if the injured person is a skipper, who could endanger the crew around them if cognitively impaired. In mariner law, the captain of a boat is legally responsible for their crew when at sea, as stated in the Marine Guidance Note 604, published by the Maritime and Coastguard Agency [33]. Thus, if a captain experiences a concussion, which results in impaired judgement, the captain could be a risk not only to himself but also the entire crew of the boat.

The SailGP Concussion Management Policy considers the unique short- and long-term risks of sailing-induced concussions and aims to ensure up-to-date guidance to improve the safety of sailing participants. It does this by encouraging baseline testing in concussed individuals, outlining the immediate actions to take if a concussion is suspected, describing the initial management of an athlete following a concussion, and proposing a programme for a graduated return to full-time sailing [Appendix A].

**Moving Forward**

Considering the unique risks of concussions in sailing, concussion prevention and education must be addressed by sailing organisations and medical professionals. Furthermore, the lack of research surrounding the topic poses a challenge in assessing the efficacy of current non-specific guidelines. To combat this, an introduction of standardised incident reporting systems should be considered to help assess the effectiveness and use of these guidelines. Moreover, coaches and race officials should have further training in concussion assessment and recognition, using sailing-specific tools such as the one developed by SailGP, modified for use by non-healthcare professionals.

## 6. Conclusions

This review highlights the gap in information surrounding sailing-specific concussion guidance. Sailing as a sport poses unique risks and barriers to the prompt diagnosis and assessment of concussions. Therefore, medical professionals and sailing organisations need to collaborate to produce sailing-specific and standardised guidelines, such as the described SailGP concussion assessment protocol. This protocol prioritises the most essential assessments while sailing, including rapid GCS, modified Maddock’s questions, and ‘on-field’ balance assessments. As a result, this allows for rapid identification of sick athletes in a high-risk, fast-paced environment. It is our recommendation that further studies be carried out comparing current guidelines and their effectiveness in assessing and diagnosing concussions, as well as following up on the success of the SailGP concussion assessment protocol, upon implementation, in rapidly determining the severity of a head injury in sailing.

## Figures and Tables

**Table 1:** Concussion guidance from sailing and sporting organisations.

Source	Type	Organisation	Year
McCrory et al. [2]	Consensus Statement	CISG (Berlin)	2017
RYA (“Sailing Concussions”) [8]	Guideline	RYA	2025
SCAT6 [13]	Guideline	CISG	2023
CRT5 [14]	Guideline	CISG	2017
Patricios et al. [15]	Consensus Statement	CISG (Amsterdam)	2023
RYA (“Pathway to Recovery”) [16]	Guideline	RYA	2025

**Figure A1:**
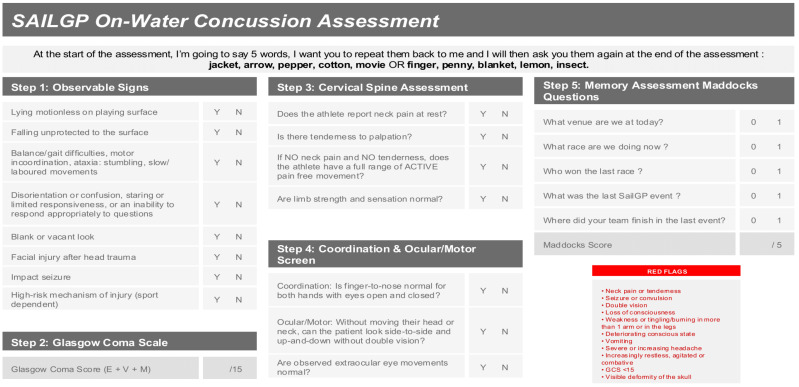

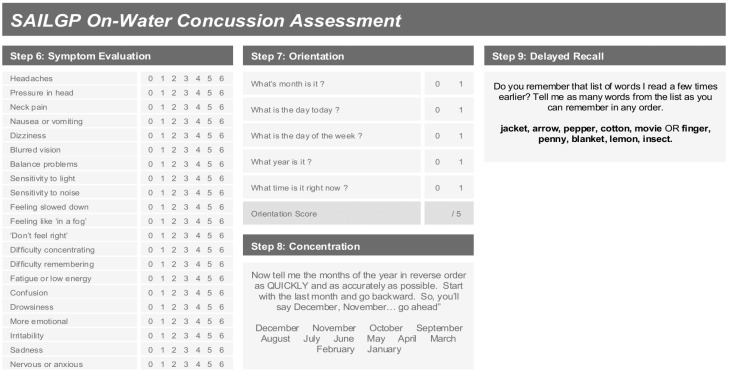
SAILGP On-Water Concussion Assessment.

## Data Availability

No new data were created or analyzed in this study. Data sharing is not applicable to this article.

## Supplementary Materials

The following supporting information can be downloaded at: https://www.mdpi.com/article/10.3390/sports13120455/s1. Supplementary File S1: PRISMA 2020 Checklist.
